# Dataset on specific UV absorbances (SUVA_254_) at stretch components of Perak River basin

**DOI:** 10.1016/j.dib.2020.105518

**Published:** 2020-04-17

**Authors:** Teh Sabariah Binti Abd Manan, Taimur Khan, Wan Hanna Melini Wan Mohtar, Salmia Beddu, Nur Liyana Mohd Kamal, Saba Yavari, Hisyam Jusoh, Sobia Qazi, Siti Khadijah Binti Imam Supaat, Fadzilah Adnan, Abdulnoor A. Ghanim, Sara Yavari, Affiani Machmudah, Armin Rajabi, Mojtaba Porhemmat, Muhammad Irfan, Mohd Tajuddin Abdullah, Elia Syarafina Binti Abdul Shakur

**Affiliations:** a Institute of Tropical Biodiversity and Sustainable Development, Universiti Malaysia Terengganu, 21300 Kuala Terengganu, Terengganu, Malaysia; b Department of Civil Engineering, Faculty of Engineering, Najran University, P.O Box 1988, King Abdulaziz Road, Najran, Saudi Arabia; c Department of Civil Engineering, Faculty of Engineering and Built Environment, Universiti Kebangsaan Malaysia, 43600 Bangi, Selangor, Malaysia; d Department of Civil Engineering, Universiti Tenaga Nasional, Jalan Ikram-Uniten, 43000 Kajang, Selangor Darul Ehsan, Malaysia; e Department of Civil and Environmental Engineering, Universiti Teknologi PETRONAS, 32610 Seri Iskandar, Perak Darul Ridzuan, Malaysia; f Department of Foundation Engineering and Physical Science, University of Nottingham, Advance Manufacturing Building, Jubilee Campus, NG8 1BB, United Kingdom; g Department of Mechanical and Materials Engineering, Faculty of Engineering and Built Environment, Universiti Kebangsaan Malaysia, 43600 Bangi, Selangor, Malaysia; h Institut de recherche en biologie végétale de l'Université de Montréal, Québec, Canada; i Faculty of Science and Technology, Universitas Airlangga, Jalan Mulyorejo, Kampus C, Surabaya City, East Java 60115, Indonesia; j Department of Electrical Engineering, Faculty of Engineering, Najran University, P.O Box 1988, King Abdulaziz Road, Najran, Saudi Arabia

**Keywords:** Perak river basin, Water, Specific UV absorbance, Total organic carbon, Mixed use area, Water pollutant

## Abstract

Perak River basin is in Perak state of Peninsular Malaysia. In this research, the river stretch serves as water intake for domestic, agricultural and industrial purposes in Perak Tengah, Hilir Perak and Manjung regions. It is located in mixed use area whilst exposing the river to anthropogenic elements. The sampling locations were conducted at selected points of Perak River namely Tanjung Belanja Bridge (TBB), Water Treatment Plant Parit (WTPP), Parit Town discharge (PTD), Water Treatment Plant Senin (WTPS) and Water Treatment Plant Kepayang (WTPK). The existence of aromatic hydrocarbons in freshwater samples was pre-assessed via qualification analysis; specific ultraviolet absorbance (SUVA_254_) method at 254 nm of wavelength. The SUVA dataset were 48.38 L/mg-m (TBB), 50.54 L/mg-m (WTPP), 8.05 L/mg-m (PTD), 85.75 L/mg-m (WTPS) and 217.39 L/mg-m (WTPK). The SUVA_254_ values of fresh water at the river basin have exceeded the water quality standards value equivalent to 2.0 L/mg-m permitted by the Environmental Protection Agency of United States. The exceeding values were an indication of a large portion of aromatic compounds in the water. Qualification analyses evident the existence of water pollutants at treacherous concentrations for public health in freshwater samples of Perak River basin. Thus, this research has presented important findings towards further research and countermeasure for a better alternative of water treatment in Malaysia.

Specifications table

**Table:** 

Subject	Environmental Science
Specific subject area	Pollution, Water Science Technology
Type of data	Figures and Graphs
How data were acquired	Instruments:
	1. UV–VIS scanning spectrophotometer (T80, Oasis Scientific Incorporation, US) with 1 cm cells at the 254 nm wavelength 2. Catalytic combustion-based TOC analyzer (TOC-5000, Shimadzu, Japan)
Data format	Raw data
Parameters for data collection	1. Total organic carbon (mg/L) 2. UV absorbances (cm^−1^) 3. SUVA_254_ (L/mg-m)
Description of data collection	1. The sampling stations were within 30.3 km of Perak River in Perak Tengah region surrounded by mixed use area. 2. Samples were analysed for total organic carbon and UV absorbances within 24 h after collection.
Data source location	Institution: Universiti Malaysia Terengganu City/Town/Region: Perak Tengah region, State of Perak Darul Ridzuan Country: Peninsular Malaysia Latitude and longitude (and GPS coordinates) for collected samples/data:] Tanjung Belanja Bridge (TBB) (N 4°30.31860′, E 100°55.50294′); Water Treatment Plant Parit (WTPP) (N 4° 29.84064′, E 100° 55.34406′); Parit Town Discharge (PTD) (N 4° 28.60158′, E 100° 54.46530′); Water Treatment Plant Senin (WTPS) (N 4° 22.95438′, E 100° 54.13446′); and Water Treatment Plant Kepayang (WTPK) (N 4° 18.94494′, E 100° 52.81428′).
Data accessibility	Data is provided in the article.

## Value of the data

• The data can be used by scientific community as research baseline for better alternative of water treatment in Malaysia and other developing countries.
• The data will benefit both local authority and public for immediate preventive measures.
• The data will give an additional value of good benchmarking for a timely improvement of Malaysian water quality standards and guidelines with an additional of parameters such as specific UV absorbances (SUVA_254_) and total organic carbons analyses.

## 1 Data description

Chemical elements, compounds and mixtures are physically and chemically unique in its properties. Some of them are carcinogenic in nature. Carcinogens in water environment can be identified accurately using an appropriate method chosen from the available alternatives. The specific ultraviolet absorbances (SUVA) method is an EPA method 415.3 [1]. It is a determination of total organic carbon and specific UV Absorbance (SUVA_254_) at 254 nm in water sample. It is calculated as the ratio of UVA_254_ (at 1 cm of the quartz cell path length) to total organic carbon (TOC) as in the following equation:

(1) $$\text{SUVA}\left(L/\text{mg}-m\right)=\frac{\text{UVA}\left(cm^{-1}\right)}{\text{TOC}\left(\text{mg}/L\right)}\times 100\;\text{cm}/m$$

SUVA can be used to estimate the percentage of aromatic carbon content of humic acid. It is also an indicator of aromaticity and chemical reactivity for aquatic organic matter samples from a wide range of water sources. However, the national standards of water quality guidelines in Malaysia [2] as well as parameter limits for sewage and industrial effluents [3] in the country are lacking such essential parameters as part of their monitoring routine.

**Fig. 1:**
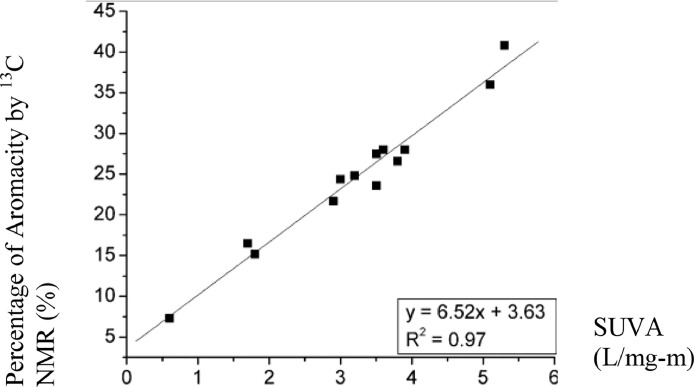
SUVA_254_ versus percent aromaticity determined by ^13^C NMR. (Source: James et al. [5]).

Nuclear magnetic resonance (NMR) is a physical phenomenon in which nuclei in a magnetic field absorb and re-emit electromagnetic radiation. ^13^C NMR is the application of nuclear magnetic resonance (NMR) spectroscopy to carbon [4]. A research on the evaluation of SUVA as an indicator of the chemical composition and reactivity of dissolved organic carbon was carried out by James et al. [5]. The data show that SUVA is significant and strongly correlated to the presence of aromatic carbon content as shown in Fig. 1.

**Fig. 2:**
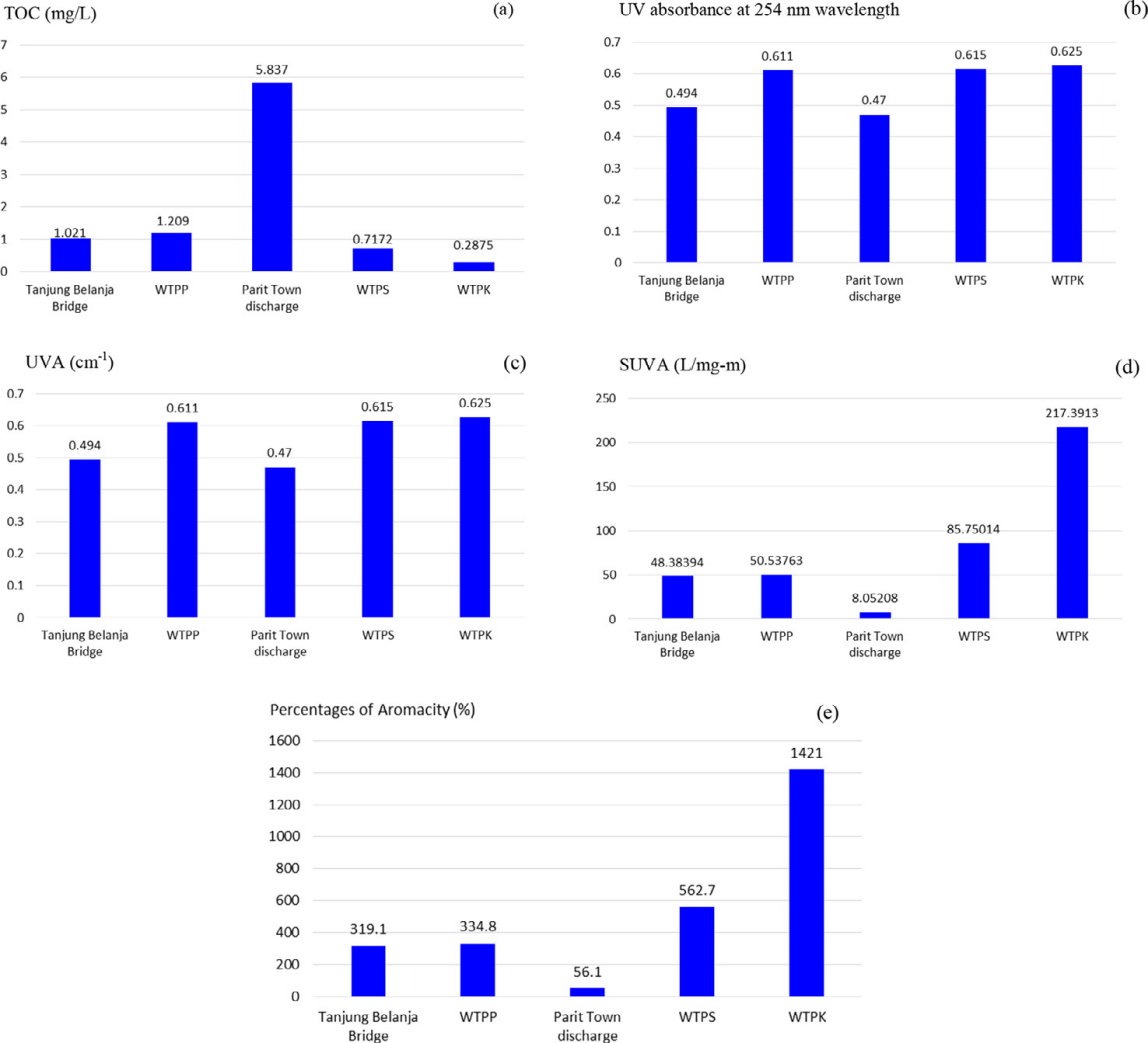
(a) Total organic carbon (mg/L), (b) UV absorbance at 254 nm wavelength, (c) UVA data (cm^−1^), (d) SUVA data (L/mg-m), (e) Percentages of aromacity (%).

The total organic carbon (TOC) data based on the river flows were 1.021 mg/L (TBB), 1.209 mg/L (WTPP), 5.837 mg/L (PTD), 0.7172 mg/L (WTPS) and 0.2875 mg/L (WTPK) (Fig. 2(a)). The data for UV absorbance at 254 nm wavelength were 0.494 (TBB), 0.611 (WTPP), 0.470 (PTD), 0.615 (WTPS) and 0.625 (WTPK) (Fig. 2(b)). The UVA data were 0.494 cm^−1^ (TBB), 0.611 cm^−1^ (WTPP), 0.470 cm^−1^ (PTD), 0.615 cm^−1^ (WTPS) and 0.625 cm^−1^ (WTPK) (Fig. 2(c)). The SUVA data were 48.38 L/mg-m (TBB), 50.54 L/mg-m (WTPP), 8.05 L/mg-m (PTD), 85.75 L/mg-m (WTPS) and 217.39 L/mg-m (WTPK) (Fig. 2(d)). Based on EPA water quality standards, SUVA monitoring data for raw water (surface water) should generally not exceed 4.0 L/mg-m. Data ranged from 5.23 L/mg-m to 217.39 L/mg-m. These are the indications of a large portion of aromatic compounds in the water. The qualification analysis in selected points of riverine environment using the specific ultraviolet absorbance (SUVA) method showed the existence of PAHs with percentage ranges from 39.2% to 100% as shown in Fig. 2(e).

## 2 Experimental design, materials, and methods

Water sampling was conducted at 5 different sampling points from Perak River namely Tanjung Belanja Bridge (TBB), Water Treatment Plant Parit (WTPP), Parit Town discharge (PTD), Water Treatment Plant Senin (WTPS) and Water Treatment Plant Kepayang (WTPK). The sampling points were used for genotoxicity and quantification analyses by Malakahmad et al. [6] and Abd Manan et al. [7]. Water samples were taken using amber bottle (1 L). These bottles were washed with phosphate-free detergent and 1 mol nitric acid (HNO_3_) and rinsed with tap water between intervals. Bottles were rinsed with water samples before filled up with sample water for collection and stored in cold room at 4 °C [8,9]. The SUVA method presented in this research was a preliminary detection on carcinogens particularly aromatic hydrocarbons in the water samples. As for further investigation for researchers, other detection methods for carcinogens such as atomic absorption spectrophotometer, gas chromatography mass spectrometry and high performance liquid chromatography analyses were briefly described and can be referred in Malakahmad et al. [10].

SUVA (EPA Method 415.3) was used in qualification analysis to determine the existence of PAHs in selected points of riverine environment [1]. TOC measurements were performed using a catalytic combustion-based TOC analyzer (TOC-5000, Shimadzu, Japan). The ultraviolet absorbance (UVA_254_) was determined using a UV–VIS scanning spectrophotometer (T80, Oasis Scientific Incorporation, US) with 1 cm cells at the 254 nm wavelength. It is calculated as the ratio of UVA_254_ to TOC as in Eq. (1). Sample was filtered using filter paper (Whatman, No. 1) before poured into spectrophotometer vial (1 ml). Ultraviolet absorbance (A/cm) was measured at 254 nm.

## Appendix Supplementary materials

Image, application 1
